# A prospective, randomized, double-blind, placebo-controlled parallel-group dual site trial to evaluate the effects of a *Bacillus coagulans*-based product on functional intestinal gas symptoms

**DOI:** 10.1186/1471-230X-9-85

**Published:** 2009-11-18

**Authors:** Douglas S Kalman, Howard I Schwartz, Patricia Alvarez, Samantha Feldman, John C Pezzullo, Diane R Krieger

**Affiliations:** 1 Miami Research Associates, 6141 Sunset Drive, Suite 301, South Miami, Florida 33143 USA; 2 Latin American Research, Av. Maximo Gomez #60, Suite 201, Plaza Paseo Del Teatro, Santo Domingo, Dominican Republic

## Abstract

**Background:**

This randomized double blind placebo controlled dual site clinical trial compared a probiotic dietary supplement to placebo regarding effects on gastrointestinal symptoms in adults with post-prandial intestinal gas-related symptoms (abdominal pain, distention, flatulence) but no gastrointestinal (GI) diagnoses to explain the symptoms.

**Methods:**

Sixty-one adults were enrolled (age 36.5 ± 12.6 years; height 165.1 ± 9.2 cm; weight 75.4 ± 17.3 kg) and randomized to either Digestive Advantage™ Gas Defense Formula - (GanedenBC^30 ^*Bacillus coagulans *GBI-30, 6086): n = 30; or Placebo: n = 31. Study subjects were evaluated every two weeks over a four-week period using validated questionnaires and standard biochemical safety testing. Outcome criteria of interest included change from baseline in Gastrointestinal Symptom Rating Scale (GSRS) abdominal pain, abdominal distention, flatus, and the Severity of Dyspepsia Assessment (SODA) bloating and gas subscores over four weeks of product use.

**Results:**

Measured against the placebo, subjects in the probiotic group achieved significant improvements in GSRS abdominal pain subscore (p = 0.046) and the GSRS total score (p = 0.048), with a strong trend for improvement on the GSRS abdominal distension subscore (p = 0.061). A strong placebo effect was evident which could explain the lack of statistical significant differences between the groups for many of the efficacy variables.

**Conclusion:**

In conclusion, the *Bacillus coagulans*-based product was effective in improving the quality of life and reducing gastrointestinal symptoms in adults with post prandial intestinal gas-related symptoms and no GI diagnoses.

**Trial Registration:**

ClinicalTrials.gov Identifier: NCT00881322

## Background

It is estimated that only 10% of the 10^14 ^cells in the human body actually belong to the body itself. The overwhelming majority of cells consist of a diverse ecology of nonpathogenic bacteria, and 1-2 kg of them live in the gut alone, mainly in the large intestine [1]. Bengmark suggests that human beings should indeed be considered to have two separate, equally vital digestive systems: one being the organs of the gastrointestinal tract; the other being the bacteria that colonize them [2]. The bacteria have defined an ecological niche for themselves in the intestines, fermenting non-digestible dietary residue and endogenous mucus from the epithelia [3]. Though the colon contains over 500 strains of bacteria, it is generally dominated by 35-40 different types of microbes. Many disorders of the gut have been associated with a disturbance in this distribution of species. Inflammatory bowel disease, diarrhea, and even multisystem organ failure [4] are believed to be correlated with an imbalance in gut ecology favoring the growth of pathogenic strains [5].

Probiotics are nutritional supplements designed to target pathogenic microbial species distribution by augmenting the growth of nonpathogenic bacteria. A commonly accepted definition of probiotic is "a preparation of or a product containing viable, defined microorganisms in sufficient numbers, which alter the microflora (by implantation or colonization) in a compartment of the host and by that exert beneficial health effects on the host [6]." There is strong evidence that probiotics work by helping non-pathogenic bacteria to compete with their pathogenic counterparts for nutrient availability as well as for adhesion sites along the intestinal lining, preventing both the overgrowth of pathogenic bacteria as well as their translocation through the epithelial mucosa into the rest of the body [7]. There is also evidence to suggest that intestinal flora play an important role in immune system response. Studies in humans and rodents have shown that probiotic treatment is directly correlated with an increase of salivary immunoglobin A (sIgA) production. Furthermore, exposure to luminal microbes instantly increases the number of intraepithelial lymphocytes.

In addition to overwhelming evidence in support of the effectiveness of probiotics, their lack of detrimental side effects is further reason for their growing popularity. In fact in a recent review paper, Levri et. al. suggest that physicians' advice to patients regarding a given probiotic should be a cavalier "try it [8]." It is no surprise then that there is great interest in investigating their use as an inexpensive treatment for a variety of causes of gastrointestinal discomfort.

Digestive Advantage™ Gas Defense Formula (Ganeden Biotech, Mayfield Heights, Ohio) is a probiotic supplement containing *Bacillus coagulans *as well as an enzyme blend of cellulases from *Trichoderma longibrachiatum *and *Aspergillus niger*. Studies suggest that the probiotic Bacillus coagulans decreases the symptoms of abdominal pain and bloating in subjects with inflammatory bowel disease [9]. With this in mind, we undertook a randomized, double-blind, placebo-controlled clinical trial to evaluate Gas Defense (GD). The purpose of the study was to compare its effect versus placebo on gastrointestinal quality of life in adults with intestinal symptoms but no GI diagnoses.

## Methods

### Experimental Design

This double-blind, placebo-controlled clinical study randomized 61 subjects at two investigative sites (Miami and the Dominican Republic). Subjects provided written informed consent prior to participating in any study procedures. Subjects were then randomized within each site in a 1:1 manner into intervention (GD) or placebo groups. Investigators and subjects were blinded to product assignment. Subjects were seen at three visits over the course of four weeks - a screening/randomization visit at Day 0, and two follow-up visits at Days 14 and 29. On Day 0, the participants were instructed to begin taking one capsule daily, at approximately the same time of day, and to continue doing so for the duration of the study. Participants were provided sufficient product at visits 1 and 2 to cover the time between visits. Compliance with product use was measured via the pill counting method. During each visit, the participants were evaluated with a series of questionnaires in addition to hemodynamics and adverse event monitoring. The research was in compliance with the Helsinki Declaration and approved by the Aspire Independent Review Board San Diego, California (approved May 13, 2008) and Consejo Nacional de Bioetica en Salud (Conabios), Santo Domingo, Dominican Republic (approved June 23, 2008).

### Subject Population

Subjects were drawn from the Greater Miami area and the Dominican Republic. All were between 20-68 years of age and had self-reported post-meal intestinal gas-related symptoms including abdominal pain, cramps, distended feeling/bloating, and flatulence. Out of a total of 98 subjects interviewed by phone, 64 attended the screening evaluation. Three of those subjects did not meet entry criteria. In the final study population, seven subjects came from Miami and 54 came from the Dominican Republic. Sixty subjects began the study but one was terminated at the discretion of the investigator after a single dose. An additional subject was subsequently enrolled with IRB notification and approval. All subjects were in otherwise good health, willing and able to comply with the protocol, and, if female, neither pregnant nor lactating and willing to use a reliable method of birth control. All subjects signed the IRB-approved Informed Consent prior to any procedures being conducted.

Exclusion criteria for entering this study included; active heart disease, uncontrolled high blood pressure, renal or hepatic impairment, Type I or II diabetes, psychiatric and immune disorders, unstable thyroid disease, Parkinson's disease, a history of cancer, previous stomach or intestinal surgery, the consumption of medication or supplements that would interfere with the natural flora of the gut such as antibiotics, probiotics, or prebiotics within the last 30 days prior to screening. Subjects with gastrointestinal disorders or other digestive problems such as Crohn's disease, short bowel, ulcerative colitis, Irritable Bowel Syndrome, constipation, or lactose intolerance were also excluded. Lactose intolerance was excluded as per subject profession or previous diagnosis. Similarly, subjects using GI medications to control the function of the gut, such as anti-spasmodics, motility agents, pro-kinetic agents, or laxatives were excluded. Subjects were only permitted to use over-the-counter gas relief products as rescue treatment during the study. Only one subject reported having done so. Subjects allergic to wheat, fish, or any other ingredients in GD or the placebo were excluded.

### Intervention

The active product tested is a probiotic supplement containing *Bacillus coagulans *(specifically *Bacillus coagulans *GBI-30, 6086, also known as GanedenBC^30^). The product specifically contained *B. coagulans*, Enzyme Blend (cellulase - *Trichoderma longibrachiatum*, cellulase - *Aspergillus niger*, hemicellulase, α-galactosidase, invertase) with the inactive ingredients of a vegetarian capsule, magnesium stearate, silicon dioxide, and maltodextrin. There were 2.0 × 10^9 ^colony forming units per capsule.

The placebo was provided by the manufacturer and matched in size and color to the active product. Independent product analysis for content was carried out to confirm label content claim (ULTRAtab Labs, Highland, New York). All subjects were instructed to take one tablet daily for the duration of the study.

### Assessment

During the study, subjects were asked to complete several questionnaires, each targeting a different symptom. Distension, pain, and flatus were tracked using the corresponding subsections of the GI Symptoms Rating Scale (GSRS) [10]. Bloating and gas were measured with the Severity of Dyspepsia Assessment (SODA) [11]. Other assessments included the GSRS overall score and the SODA Non-Pain Symptoms (NPS) subscore, as well as the SODA subscore for satisfaction with dyspepsia-related health, SF-36v2 quality of life physical and mental component summaries, and 7-point anchored Visual Analog Scale (VAS-Gas) assessment of gas symptoms.

All questionnaires were completed by the study subjects at every visit, except for VAS-Gas, which was administered only at the second and third visits because it asks for a consideration of relative change from baseline. Blood pressure and heart rate were measured at each visit, and study compliance was monitored by the pill count method.

### Statistical Methods

The two primary endpoints for analysis in this study were the GSRS subscores for abdominal pain, distension, and flatus; and the SODA subscores for bloating and gas. Other endpoints included the GSRS overall score, SODA-NPS score, SODA subscore for satisfaction with dyspepsia-related health, the SF-36v2 summaries, and the VAS-Gas assessment.

The formal efficacy analysis consisted of a set of analyses of covariance (ANCOVAs), one for each efficacy endpoint. The value of the efficacy variable at Visit 3 (end of study) was the dependent variable, the product group (GD or placebo) was the variable of interest, and the value of the efficacy variable at Visit 1 (baseline) was a covariate. Investigative site (US or DR) was also included in the model. Only p-values less than or equal to 0.05 were considered significant.

Other descriptive (non-inferential) summaries and comparisons were carried out - mean changes from baseline to each subsequent time point were tested by the paired Student t test or Wilcoxon signed-ranks test, and mean differences between product groups were tested by the unpaired Student t test or Mann-Whitney U test. Differences in the distribution of categorical variables between the product groups were tested by the Fisher Exact test.

Sample size was determined on the basis of time, cost, and the ability to detect a clinically important effect size. It was determined that 25 analyzable subjects per group would provide 80% power to obtain a significant result for a 0.8-sigma effect size. To allow for a possible 15% attrition from the study, 30 subjects were enrolled per group. No adjustment for multiple testing was applied in the analysis of data from this study. Each test was evaluated at the 0.05 alpha level (p ≤ 0.05 considered significant).

## Results and Discussion

Most subject characteristics at baseline (the screening/randomization) were evenly matched between the two product groups (Table 1). The placebo group was, on average, four years older and eight kilograms heavier than the GD group. Most of the endpoints tracked did not show a significantly different response between GD and placebo. These included the GSRS increased flatus subscore, the SODA bloating subscore, the SODA non-pain symptoms and satisfaction scores, and the SF-36v2 physical and mental component summaries. However, all but the SF-36v2 MCS showed differences in the direction that indicated a larger beneficial effect for GD than the placebo.

**Table 1:** Baseline and Descriptive Characteristics

Group	Gas Defense (n = 30)	Placebo (n = 31)
**Site**		
Dominican Republic	27 (90%)	27 (87%)
Miami, FL	3 (10%)	4 (4%)

**Age**, Years	34.8 ± 12.5	38.2 ± 12.6

**Gender**		
Female	16 (53%)	17 (55%)
Male	14 (47%)	14 (45%)

**Ethnicity**		
Hispanic	27 (90%)	28 (90%)
Non-Hispanic	3 (10%)	3 (10%)

**Race**		
Black/AA	8 (27%)	7 (23%)
Caucasian	9 (30%)	9 (29%)
Other	12 (43%)	15 (48%)

**Height**, cm	164.2 ± 8.6	165.8 ± 9.8

**Weight**, kg	71.4 ± 14.1	79.2 ± 19.3

**Status**		
Completed Protocol	30 (100%)	30 (97%)
Early Termination	0 (0%)	1 (3%)

**Heart Rate**, beats/minute	69.9 ± 12.1	70.7 ± 10.3

**Systolic Blood Pressure**, mm Hg	121.2 ± 17.0	122.2 ± 10.9

**Diastolic Blood Pressure**, mm Hg	75.1 ± 9.0	76.0 ± 7.2

**GSRS - Abdominal Pain Subscore**	3.17 ± 1.85	3.14 ± 1.48

**GSRS - Abdominal Distension Subscore**	3.38 ± 2.13	4.14 ± 1.43

**GSRS - Increased Flatus Subscore**	3.86 ± 1.92	4.07 ± 1.53

**GSRS - Total GI Symptom Score**	40.8 ± 19.8	39.4 ± 12.1

**SODA - Bloating Subscore**	2.52 ± 1.48	2.93 ± 1.25

**SODA - Gas Subscore**	3.28 ± 0.96	3.28 ± 0.84

**SODA - Non-pain Symptoms Score**	16.83 ± 3.35	17.00 ± 2.09

**SODA - Satisfaction Score**	8.3 ± 3.4	9.2 ± 3.5

**SF-36v2 - Physical Component Summary**	49.9 ± 8.5	49.0 ± 9.9

**SF-36v2 - Mental Component Summary**	51.3 ± 10.1	51.3 ± 9.9

Values are expressed as mean ± standard deviation.

Table 2 shows the ANCOVA coefficient of the product group - an estimate of the amount by which the four-week improvement in the GD group exceeds that of the placebo group, along with its standard error and p-value indicating whether or not there is a statistically significant difference between the product and placebo. GD performed significantly or nearly significantly better than placebo for the following endpoints (Tables 3, 4 and 5): GSRS: abdominal pain subscore (p = 0.046), GSRS: abdominal distension subscore (p = 0.061), and GSRS total score (p = 0.048).

**Table 2:** Efficacy Analysis (ANCOVA)

Endpoint	Coefficient ± Std Err	p-value
GSRS: Abdominal Pain Subscore (lower is better)	-0.627 ± 0.307	0.046 †

GSRS: Abdominal Distension Subscore (lower is better)	-0.572 ± 0.299	0.061 ‡

GSRS: Increased Flatus Subscore (lower is better)	-0.511 ± 0.353	0.154

GSRS: Total Score (lower is better)	-4.806 ± 2.381	0.048 †

SODA: Bloating Subscore (lower is better)	-0.229 ± 0.216	0.294

SODA: Gas Subscore (lower is better)	-0.348 ± 0.219	0.118

SODA: Non-Pain Symptoms Score (lower is better)	-1.025 ± 0.870	0.244

SODA: Satisfaction Score (lower is better)	-0.058 ± 1.358	0.966

SF-36v2: Physical Component Summary (higher is better)	0.941 ± 1.118	0.403

SF-36v2: Mental Component Summary (higher is better)	-2.400 ± 2.010	0.238

† Significant (p ≤ 0.05)

‡Approaches significance (p ~ 0.05)

**Table 3:** Gastrointestinal Symptom Rating Scale Abdominal Pain Subscore

Visit	Gas Defense	Placebo
Day 0 Screen/Rand	3.17 ± 1.85 (29)	3.14 ± 1.48 (29)

Day 14 Mid-Study	2.10 ± 1.29 (29)	2.28 ± 1.51 (29)

Day 29 End-of-Study	1.59 ± 0.95 (29)	2.21 ± 1.45 (29)

Change from Day 0 to Day 14	-1.07 ± 1.70 (29)	-0.86 ± 1.81 (29)

Change from Day 0 to Day 29	-1.59 ± 1.70 (29)	-0.93 ± 1.67 (29)

Values are expressed as mean ± standard deviation.

**Table 4:** Gastrointestinal Symptom Rating Scale Abdominal Distension Subscore

Visit	Gas Defense	Placebo
Day 0 Screen/Rand	3.38 ± 2.13 (29)	4.14 ± 1.43 (29)

Day 14 Mid-Study	1.83 ± 1.04 (29)	2.48 ± 1.38 (29)

Day 29 End-of-Study	1.66 ± 1.08 (29)	2.38 ± 1.21 (29)

Change from Day 0 to Day 14	-1.55 ± 1.88 (29)	-1.66 ± 1.70 (29)

Change from Day 0 to Day 29	-1.72 ± 2.02 (29)	-1.74 ± 1.68 (29)

Values are expressed as mean ± standard deviation.

**Table 5:** Gastrointestinal Symptom Rating Scale Total Score

Visit	Gas Defense	Placebo
Day 0 Screen/Rand	40.8 ± 19.8 (29)	39.4 ± 12.1 (29)

Day 14 Mid-Study	29.0 ± 8.7 (29)	31.6 ± 11.4 (29)

Day 29 End-of-Study	25.2 ± 10.0 (29)	29.4 ± 9.7 (29)

Change from Day 0 to Day 14	-11.9 ± 16.4 (29)	-7.8 ± 11.7 (29)

Change from Day 0 to Day 29	-15.6 ± 17.4 (29)	-9.9 ± 12.3 (29)

Values are expressed as mean ± standard deviation.

While other efficacy endpoints do not indicate statistical significance for GD relative to placebo, all but the SF-36v2 MCS and the VAS-Gas score showed differences in the direction that indicates a larger beneficial effect for GD than for placebo.

The lack of significance for many of the efficacy endpoints can be attributed to several factors. First, as seen in the descriptive summary tables for each endpoint, a very strong placebo effect was evident in this study. Subjects generally liked the product they were taking and tended to report substantial improvement regardless of which product they were taking. All endpoints showed large four-week improvement in both product groups. This may be partly cultural, with people wanting to demonstrate what they considered to be the "expected" improvement, although this cannot be established from the available data. Whatever the cause, this kind of phenomenon is quite common in studies involving subjective endpoints (especially discomfort-related endpoints). With the placebo group showing such a large improvement, there was not much "room for improvement" for the GD group over placebo.

Also, there was a considerable amount of random variability in most of the efficacy endpoints. That is, most of the efficacy variables had large within-group standard deviations for four-week changes from baseline. This is quite common with subjective, semi-quantitative endpoints like the GSRS and SODA questionnaire scales, and it has the effect of reducing the power to detect significance. This study was powered to provide a good chance of getting a significant result for an endpoint if the average amount of improvement (for GD, compared to Placebo) was at least 4/5 as large as the within-group standard deviation for that endpoint. In this study, the magnitude of the improvements tended to be less than that.

## Conclusion

The *Bacillus coagulans*-based probiotic product showed superior numerical scores to placebo in 10 of 12 efficacy variables, and the differences were significant or nearly significant in three of the 12 variables. Within this study population, the *Bacillus coagulans*-based probiotic product was effective and safe for abating symptoms of gastrointestinal distress, particularly abdominal pain and distention in the post-prandial period.

## Competing interests

The authors received funding for this study from Ganeden Biotech.

## Authors' contributions

DSK participated in the design of the study and drafting of the manuscript. HIS participated in the design of the study and served as a Sub Investigator. PA participated in the design of the study, served as co-Principal Investigator and contributed to the manuscript. SF participated in the design of the study, data collection and manuscript preparation. JCP participated in the study design, data collection and performed the statistical analysis. DRK participated in the design of the study, served as co-Principal Investigator and contributed to the manuscript. All authors read and approved the final manuscript.

## Pre-publication history

The pre-publication history for this paper can be accessed here:

http://www.biomedcentral.com/1471-230X/9/85/prepub
